# Obstetrical Auscultation

**Published:** 1852-10

**Authors:** M. M. Rodgers

**Affiliations:** Rochester, N. Y.


ART. II.—*Obstetrical Auscultation. Signs of Pregnancy.* By M. M. RODGERS, M. D., Rochester, N. Y.

It is not designed, in this brief article, to describe in detail, all the signs of pregnancy, or to consider them in the order of their relative value. We shall only notice briefly those signs which usually accompany utero-gestation, and which in the aggregate furnish strong presumptive evidence of this condition. As these signs, however, are all more or less equivocal, whether taken individually or collectively, we propose to consider the value of those results furnished by auscultation. If, by this mode, we are able to arrive at a sign, which, taken alone, and independently of all others, will at all times give unequivocal *positive* evidence,—its importance in a medico, legal, moral, and scientific point of view will be admitted by all. The signs upon which we have formerly been accustomed to rely for a diagnosis in suspected pregnancy, may be noticed, for the purpose of showing, not what they indicate, but what they do *not indicate*, for their evidence is entirely negative.

---

1. *The general condition* of a woman *enceinte*, may lead to this suspicion, especially if she be primiparous. 2. The *cessation of the menses*, but there are so many exceptions to this, that it cannot be relied upon: menstruation may continue in cases of pregnancy, until nearly the close of the term, and may cease in women not pregnant. 3. The *morning sickness*, which occurs usually, between the sixth and twelfth week, is often absent. 4. *Salivation* occurs in some cases, but may arise from other causes, and is not often present. 5. *Enlargement of the mammæ*, is a pretty constant sign, but is occasionally absent, and may occur under other circumstances also. 6. *The areola and enlargement of the follicles*, are also nearly constant, but occur under other circumstances. 7. *Secretion of milk* usually takes place during the latter half of the term, but not always: it may occur also, in women not pregnant, and even in girls and men. 8. *Increased size of the abdomen*, when taken in connection with ohter signs, is of value, but it may be a consequence of disease also. 9. *Condition of the umbilicus*, is a sign of little value. 10. *Dullness on percussion*, over the abdomen must occur in pregnancy, but may be found in other conditions. 11. *Quickening*, or the motions of the fœtus, usually occurs at the end of the fourth month, and is a very constant sign, but it may sometimes be produced by the voluntary or involuntary action of the abdominal muscles, and is sometimes never felt, in cases of real pregnancy. 12. *Ballottement*, next to auscultation, furnishes the most unequivocal evidence, and is considered by some authors, infallible. But, on the authority of Prof. Depaul, of Paris, it has led to the error of pronouncing a hydatid tumor a case of pregnancy. In cases of twins, and where there is a small quantity of amniotic fluid, it is sometimes impossible to obtain this result. 13. *Violet color of the vagina*, is very generally present in pregnancy; but the writer has seen this test made extensively in Paris, when it occasionally failed both ways. 14. *Changes in the uterus*, may occur similar to those of pregnancy, from disease. 15. *Buffy coat on the blood*, is considered by some authors a sign worthy of confidence, but this occurs in so many diseases of both sexes that it must be of little value as a test. 16. *The urine* is said to contain an unusual quantity of uric acid in pregnancy, but this occurs also in diseases of both sexes. 17. *Kiesteine* is usually present in the urine of pregnant women, but is also found in the urine of men and children, as a result of peculiar diet and disease. 18. *Palpation* of the abdomen sometimes affords very strong evidence of the presence of a fœtus, but is seldom reliable alone. 19. Besides these, there are several minor signs which are of some value, considered with the others: such are, variations of the pulse, the appetite, maculæ on the face, vaginal secretions, venereal desires, organic sympathies, mental conditions, temper, age, presence of the hymen, certain diseases, &c.

Now we see that these signs, taken together or singly, yield equivocal evidence, — evidence which at best is only negative. We want a sign which will, in all cases where it is present, give positive proof; and this sign is furnished by auscultation.

The *bruit placentaire*, is an intermittent, whizzing sound, resembling the *bruit de soufflet* of the heart, and synchronous with the maternal pulse. It may usually be heard from the end of the second month of utero-gestation, until the last pains of labor. This sound is sometimes simulated by the ovarian vessels, the uterine sinuses, abdominal vessels, the vessels of fibrous tumors and aneuresmal varix; but it need not be confounded with any but the latter sound. This sound is now supposed to be in the uterus, and not, as formerly, in the placenta; it does not, when present, indicate pregnancy positively,—nor when a fœtus is present, does it indicate its life, as it may continue for sometime after death takes place. The *funis soufflet*, may sometimes be heard intermitting synchronously with the fœtal pulsation,— but it cannot exist independently of the action of the fœtal heart, and when this can be detected it is of no use in diagnosis. The *fœtal tic tac*, or pulsation of the fœtal heart, consists of short, double, regular pulsation, resembling those of the new born infants, varying in velocity from 120 to 140 in a minute. This sound cannot be simulated by, nor confounded with any other; so, when it can be distinctly heard, it is proof *positive*, and the only one, of the presence of a living fœtus; where there is the *tic tac*, there must be a heart to produce it, and where there is a heart, there must also be a fœtus. Its absence proves only negatively, that there is no fœtus, or if any, that it may be dead.

There is no known sign by which we can determine that a woman is not pregnant. This fact makes this sign the more valuable, as in nearly all cases of actual pregnancy with a living fœtus, we may at once verify it. The fœtal *tic tac*, according to different authors, may be heard from three and a half to five months after conception. The location of the sound and the manner of obtaining it, we need not indicate.

This sign alone furnishes the means of diagnosing twin pregnancy, determining any thing in relation to the fœtal health, or the presentation to be expected when labor commences. If, then, this is a true test, as we assume, how do those physicians appreciate its value, who never auscult the abdomen at all? What confidence ought to be placed in the testimony of a medical witness in court, who should base his opinion of pregnancy entirely upon those signs, every one of which he knows to be equivocal? No matter what may be said of ballottement, aggregate signs, age, experience, learning, they all vanish like vapor before the sunbeam, when compared with this. We may be pardoned then, if we exhort those who wish to be considered "read up," and who still entertain "peculiar views," to *study obstetrical auscultation by the bedside, study it, where alone it can be learnt, on the abdomen of woman.*

